# Different Gene Expressions of Alpha and Beta Glucocorticoid Receptors in Asthmatics

**Published:** 2018

**Authors:** Kayvan Saeedfar, Mehrdad Behmanesh, Esmaeil Mortaz, Mohammad Reza Masjedi

**Affiliations:** a *Department of Genetics, Faculty of Biological Sciences, Tarbiat Modares University, Tehran, Iran.*; b *Tracheal Diseases Research Center, National Research Institute of Tuberculosis and Lung Diseases (NRITLD), Shahid Beheshti University of Medical Sciences, Tehran, Iran. *; c *Department of Immunology, Faculty of Medicine, Shahid Beheshti University of Medical Sciences, Tehran, Iran. *; d *Airways Disease Section, National Heart and Lung Institute, Imperial College London, London, UK. *; e *Tobacco Control Research Center, Iranian Anti-Tobacco Association, Tehran, Iran.*

**Keywords:** Asthma, Severe asthma, Glucocorticoid receptor, Gene expression

## Abstract

The response to glucocorticoids (GCs) therapy classifies severe refractory asthma (SRA) and mild asthma, so the glucocorticoid receptors (GCRs) gene expression may be involved in SRA pathogenesis. Thus, it is aimed to compare the expression levels of two GCR isoforms (GCRα and GCRβ) in SRA, mild asthmatics, and healthy controls. Total RNA was isolated from the peripheral blood mononuclear lymphocytes of 13 SRA patients, 14 mild asthma patients and 30 healthy volunteers. The expression levels of GCR isoforms were evaluated using quantitative real-time polymerase chain reaction (qRT-PCR). The expression level of GCR isoforms did not show any significant difference between the cases/control groups. However, the relative expression analysis between asthma/control, SRA/control and SRA/asthma groups was in the order of 0.933, 0.768 and 0.823 for GCRα and 0.697, 1.014 and 1.454 for GCRβ, respectively. Also, the expression fold change of GCRα/GCRβ in asthma, SRA and control groups was 786.88, 445.72 and 588.13, respectively. The GCRα and GCRβ isoforms did not show any correlation in SRA; but they had significant correlation in both healthy volunteers (r = 0.490, *P* = 0.007) and mild asthmatics (r = 0.786, *P* = 0.001). Also, the GCRα expression level had significant inverse correlation with age in SRA (r = -0.709, *P* = 0.007). Glucocorticoid receptors are related to, but not directly responsible for GC resistance. Since the GCRα/GCRβ expression ratio decreased in SRA, studies are needed to assess its value in diagnosing GC resistance.

## Introduction

Asthma, a chronic inflammatory respiratory disease, is characterized by recurrent attacks of breathlessness and wheezing, which vary in severity and frequency from person to person. The pathophysiology of asthma is not completely understood; but is now believed that the numerous environmental causative and/or trigger agents (such as allergens, tobacco smoke, chemical irritants, microorganisms, *etc.*) may interact with individual genetics and epigenetic backgrounds to cause asthma or induce its attack (1).

The World Health Organization (WHO) estimates show that more than 300 million people currently suffer from asthma worldwide and this number is expected to increase to 400 million by 2025. Nearly 250,000 premature deaths occur annually due to asthma. Asthma continues to be a major cause of disability and poor quality of life. It is also a major cause of health resource utilization and a public health concern worldwide (2, 3).

The definition and classification of severe asthma, which comprises nearly 5-10% of asthmatics (4), has been under debate during the past decades. However, the Innovative Medicine Initiative (IMI) published an international consensus statement and clarified a unique definition, classification, and diagnostic algorithm for severe refractory asthma (SRA) as follows:

“The term ‘severe refractory asthma’ should be reserved for patients with asthma in whom alternative diagnoses have been excluded, comorbidities have been treated, trigger factors have been removed (if possible) and compliance with treatment has been checked, but still have poor asthma control or frequent (≥2) severe exacerbations per year despite the prescription of high-intensity treatment or can only maintain adequate control when taking systemic corticosteroids and are thereby at risk of serious adverse effects of treatment” (5).

Glucocorticoids (GCs) are among the mainstays of treatment in asthmatics (1). Classification of asthmatics to mild or severe is mainly based on response to GCs (especially the inhaled forms). While systemic GCs are recommended for treatment of severe asthma (4), steroid resistance is being recognized increasingly (6).

Glucocorticoids exert their effects mainly via binding to their intracellular receptors (GCRs). So, among the proposed mechanisms of GCs resistance, the possible role of GCRs in SRA development is prominent (7-9). The resistance varies with disease type, drug regimen, and genetic background, which later may occur due to some mechanisms such as encoding gene polymorphism (*i.e.* *NR3C1* gene), altered expression level of the GCR gene, GCR modification, effects of other transcription factors and defective histone acetylation (10-12).

Human GCRs are proteins coded by the nuclear receptor subfamily 3 group C number1 (*NR3C1*) gene on chromosome 5q31.3. The GCRα is one of the four known GCR isoforms generated by alternative splicing. It has been detected in all tissues and binds to GCs. Then, the GCRα-GCs complex translocate to the nucleus and implements its role by two mechanisms. First, as a transcription factor, it binds to promoter regions and down regulates many inflammatory genes (*e.g.* *IL-1*, *IL-2*, *IL-4*, *IL-6*, *IFNγ*, *ICAM-1*, *VCAM-1*, *NO synthase*, *etc.*) or up regulates anti-inflammatory genes (*e.g.* *Lipocortin 1*, *IκBα*, *etc.*). Second, the complex may interact with other transcription factors like AP-1, NF-AT, NF-κB or STAT family members (13, 14). The other isoform, *i.e.* GCRβ, has been found in different, but not all, tissues and does not bind to glucocorticoids; so it is transcriptionally inactive (12, 15 and 16). However, it seems that GCRβ may inhibit the effect of GCRα-GCs complex in a concentration-dependent manner (17). The increase in GCRβ expression in steroid resistant conditions indicates that it is associated with GC resistance (4, 6, 17 and 18). Moreover, increased immunoreactivity of GCRβ has been demonstrated in bronchoalveolar lavage (BAL) cells, airways tissues (epithelial and submucosal inflammatory cells) and also peripheral blood mononuclear lymphocytes (PBMLs) of SRA patients (19-21). Furthermore, Pietraz *et al.*, and Panek *et al.* reported the association of NR3C1 gene polymorphisms with asthma and severe asthma in the Polish population (22, 23). However, previous studies, especially limited *in-vivo* studies, have not proven that these receptors can directly cause GC resistance (15). Therefore, controversy regarding these peptides underlies the necessity of evaluation of their accurate expression in clinical cases. Moreover, two other GCR isoforms (*i.e. *GCRγ and GCRδ) are less understood (24, 25).

Accordingly, as the difference in GCR expression levels is a proposed hypothetical mechanisms for occurrence of GC resistance, this study was performed to evaluate the expression level of GCRα and GCRβ (from *NR3C1* gene) in PBMLs of SRA patients, in order to investigate differences among asthmatics and healthy volunteers.

## Experimental

This cross-sectional study was a joint investigation undertaken by the Tarbiat Modares University (TMU) of Tehran and the National Research Institute of Tuberculosis and Lung Diseases (NRITLD) from 2012 through 2014.

*Case Selection and Sampling*

Thirteen SRA patients were selected sequentially from asthma clinic of NRITLD, according to the criteria mentioned in the 2011 international consensus for definition of severe asthma (5). The cases were visited by at least one certified pulmonologist. In addition, 14 non-severe asthma patients were enrolled in the study by the same physicians and clinic, according to the criteria of the Global Initiative for Asthma (GINA) (1). 

The 30 volunteer participants in the control group had no clinical or paraclinical history of disorders at the time of the study. Demographic and clinical data gathering, together with blood sampling, were performed by a trained expert clinician. The blood samples were immediately incubated at 4 °C for up to two h prior to lab procedures.

All of the participants voluntarily agreed to enroll in the study and signed written informed consent forms. Their demographic and clinical data were kept confidentially and there was no intervention to be applied throughout the clinical management of the cases. The ethical committee of the NRITLD approved all stages of the study under the code sbmu1.REC.1391.1 dated May 14, 2012.

*RNA Extraction and cDNA Synthesis*

After drawing 5 milliliters of venous blood, PBMLs were separated by Lympholyte^®^-H (Cedarlane Co., Burlington, Ontario, Canada) solution based on its relevant protocol. Using RNX^Plus^ solution (CinnaGen Co., Tehran, Tehran, Iran), total RNA was isolated according to the manufacturer’s instructions and kept at -80 °C until molecular analysis. The quality and quantity of the extracted RNA were checked by agarose gel electrophoresis and spectrophotometry, respectively.

Using Oligo-dT, random hexamer and reverse transcriptase enzyme (Fermentas, Thermo Fisher Scientific Co., Waltham, Massachusetts, USA), cDNAs were synthesized from 3 micrograms of isolated RNAs. The prepared cDNAs were checked by simple PCR on glyceraldehyde 3-phosphate dehydrogenase (*GAPDH*) gene and the qualified ones were kept at -80 °C to be studied by qRT-PCR.

*Primer Design and qRT-PCR*

The designed primers for amplification of GCRα and GCRβ and their specifications are shown in Table 1. Unfortunately, similar to other studies (26), it was not technically possible to design a pair of primers to distinguish the two variants GCRα and GCR-γ by qRT-PCR, because GCR- γ has only three more sequential nucleotides (TAG, codes for arginine) after nucleotide 1844 of the GCRα mRNA variant (27, 28). In order to normalize expression of our desired genes (GCRα and β), the expression of the housekeeping gene of *GAPDH* was evaluated simultaneously by qRT-PCR (as the endogenous control) for each sample (Table 1).

Using an Applied Biosystems 7500 sequence detection system (Applied Biosystem, Foster City, CA, USA) and Power SYBR Green I PCR Master Mix (Takara, Japan), qPCR was done according to the manufacturer’s protocol. The qRT-PCR was started by a preliminary denaturing stage at 95 °C for 15 min followed by 40 cycles of denaturation at 95 °C for 15 sec, annealing at 60 °C for 20 sec and extension at 72 °C for 20 sec.

*Statistical Analysis*

The mean Ct of at least duplicate tests for each sample was used to make a comparative analysis of gene expression for GCRα, GCRβ and GAPDH genes in all three groups of participants. The difference of the mean Cts between the desired genes of GCRα or GCRβ and GAPDH gene has been defined as delta Ct (ΔCt), which was used for further analysis. Independent sample *t*-test and one-way ANOVA with a Tukey’s HSD post-test were used to seek statistical differences between the mean ΔCts of the asthma, SRA, and control groups. Also, considering the optimal efficacy of the designed primers (97.2% for GCRα and 94.3% for GCRβ), the 2^-ΔΔCt^ method (29) was used to study any fold change in gene expressions. The Pearson’s test was used to evaluate correlations. Statistical analysis was done using the Statistical Package for Social Sciences (SPSS) Version 21.0 (Microsoft, Chicago, IL, USA) at a *p*-value of 0.05.

## Results

*Participant Characteristics*

Demographics of subjects are summarized in Table 2. Although it was desired to have matched groups, due to voluntary case selection, some inevitable differences were seen; *i.e. *ANOVA (Tukey’s HSD) test showed that the mean age of the control group was significantly different from that of asthma and SRA groups (*P* = 0.001 and *P* = 0.02, respectively) and males participated more in the control group. The body mass index (BMI), which may represent a general index for nutritional condition and physical health of participants, had no significant differences among the three groups. The ethnicity of participants was nearly the same in the three groups (60% Fars, 8% Turks, 12% Lores and 20% Kurds).

*Comparison of Gene Expressions*

**Figure 1:**
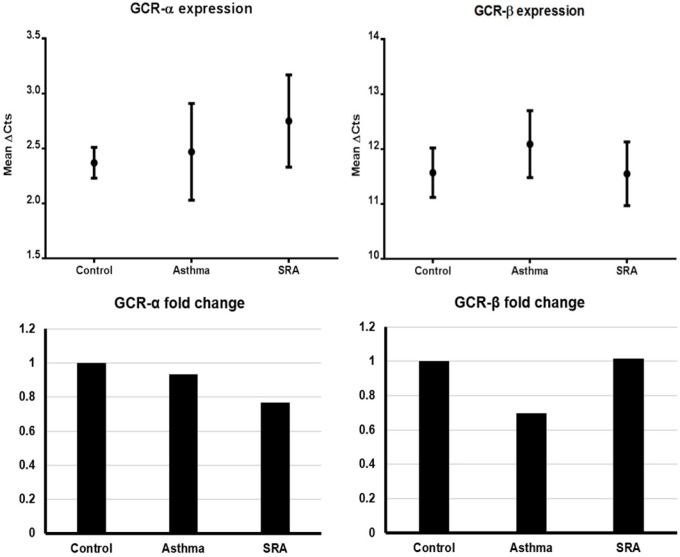
The GCRα and GCRβ expression levels and their fold changes in the three groups. Left up: GCRα expression, Right up: GCRβ expression, Left down: GCRα fold change, Right down: GCRβ fold change. (Note: the controls are adjusted to 1 in fold change graphs

Based on the results of the test of homogeneity of variances, Tamhane’s and Tukey’s HSD ANOVA tests were used to analyze GCRα and GCRβ gene expressions (mean ΔCts), respectively. In general, GCRα and GCRβ expressions were not significantly different between genders. As indicated in Table 3 and Figure 1, there were no significant differences in gene expressions among the three groups for both isoforms. The 2^-ΔΔCt ^values (*i.e. *expression ratio) of asthma/control, SRA/control and SRA/asthma groups for GCRα were in order of 0.933, 0.768, and 0.823. Similarly, these values for GCRβ were calculated to be 0.697, 1.014, and 1.454, respectively. These findings indicated that GCRα expression in SRA was lower than controls and asthma cases (ratio < 1) and vice versa for GCRβ (Figure 1). On the other hand, the expression fold changes between the GCR isoforms in asthma, SRA, and control groups were calculated to be 786.88, 445.72, and 588.13, respectively. It means the GCRα/GCRβ expression ratio in asthmatics was nearly 1.8 times that of SRA cases (786.88/445.72) and almost 1.3 times that of controls (786.88/588.13). Also, this ratio in severe asthma cases was lower than controls (detailed calculations in Supplementary file).

*Correlation with Gene Expressions*

There was no significant correlation (Pearson’s test) between age of the participants and the ΔCts of GCRα (r = -0.314, *P *= 0.17) and GCRβ (r = 0.003, *P* = 0.98). Additionally, Table 4 shows the correlation of age and BMI with GCRα and GCRβ gene expressions (ΔCts) in the mild asthma, severe asthma, and healthy groups. Although, GCRα ΔCts had non-significant mild positive correlation with age in controls; it was moderately correlated (negative, near to significant) in mild asthma group and had significant negative high correlation in SRA group. Meanwhile, the age did not show any similar pattern of correlations with GCRβ ΔCts. The correlations of GCRα ΔCts and GCRβ ΔCts were different in the three groups. Their ΔCts were significantly correlated in both healthy volunteers (r = 0.490, *P* = 0.007) and mild asthmatics (r = 0.786, *P* = 0.001). However, there were no significant correlation between the expression of GCRα and GCRβ isoforms in SRA. The dot plot diagrams are presented in Supplementary file.

## Discussion

The underlying mechanisms of GC resistance are poorly understood and may vary with disease type, treatment regimen, and the genetic background of the patient. However, some reports indicate that changes in the relative expression levels of alternatively spliced variants of GCR (like GCRβ) may account for GC resistance (4, 12).

The detailed differences in structure, function, and localization of the GCRα and GCRβ explain why GCRβ, in contrast to GCRα, does not bind to ligand (GC) and cannot affect gene expression by itself. Instead, as a dominant negative, GCRβ may represses the transcriptional activity of GCRα (12). While some experiments denote that changes in relative expression of GCRα and GCRβ may develop GC resistance in diseases (30, 31), they do not prove that the higher expression of GCRβ is directly a causative factor of GC resistance (12). In this study, we aimed to determine the relative expression of GCRα and GCRβ in the PBMLs of healthy controls, mild asthmatics, and severe asthmatics.

Totally for all participants, the GCRα and GCRβ expressions (ΔCts) were neither associated with age nor significantly different in sex groups. So, it seems that the significant difference of age and gender between the control and case groups (asthma and SRA) did not affect the interpretation of the results.‌Also, the lack of correlation between BMI and other variables (Table 4) shows that participants’ physical conditions did not affect the results.

The steroid regimens and pulmonary function evaluations of all cases were variable (during the time period, by methods, *etc.*); hence, there was the possibility of confounding in the results. Thus, these factors were not directly included in the analysis. Furthermore, to prevent bias, it was defined that the cases had to have the sampling criteria (according to GINA for asthma and IMI for SRA) for more than two years. Also, the cases were clinically controlled by the least corticosteroid regimens for at least two months. On the other hand, it seems that prescription of Inhaled Corticosteroids (ICS) had negligible systemic effects (32) while prescription of systemic corticosteroids only in SRA cases may alter GCR expressions. Thus, we suggested that in current study, the ICS prescription in all studied cases had minimal systemic effects on GCR expression of PBMLs, while systemic corticosteroids, inevitably altered GR expression in SRA.

Our results showed that age and GCRα ΔCts were negatively correlated in asthma cases (more in SRA), while they were not correlated in controls. This means that direct correlation between age and GCRα expression, seen in asthmatics, increased with disease severity. Also, correlation between GCRα ΔCts and GCRβ ΔCts in asthmatics was higher than in controls; but this correlation was not seen in SRA. Thus, some affecting factors (*e.g.* medications, other cellular pathways, *etc.*) may interfere in the mechanism of GCR expression in mild and severe asthmatics.

The preliminary review of our findings showed that there were no significant differences in GCRα and GCRβ expressions (mean ΔCts) among the cases and controls. Meanwhile, more detailed analysis by 2^-ΔΔCt^ method indicated the followings.

The comparison of GCRα fold changes (expression ratios) between the ratios of asthma/control (0.933), SRA/control (0.768), and SRA/asthma (0.823) showed that GCRα mRNAs are produced in decreasing order in controls , asthma, and SRA. Also, these figures for GCRβ were 0.697, 1.013, and 1.453, respectively. It means the decreasing order of GCRβ expression is SRA, control, and asthma (Figure 1). The latter means that GCRβ nearly expresses 1.5 times more in SRA than in asthma. Also, it was found that the GCRα/GCRβ expression ratio decreases in the order of asthma, control, and SRA groups (786.88, 588.13 and 445.72 respectively).

These figures show that a decrease in GCRα expression occurred in all asthma cases, and even more in SRA. Meanwhile, GCRβ expression increased in SRA and decreased in asthma, compared to the controls. The reduction of GCRα expression and rise in GCRβ expression in SRA group (in comparison to controls) explain the decrease of GCRα/GCRβ expression ratio; the findings that have been showed by IHC study previously (21). But for asthma, it seems that the decrease in GCRβ expression is greater than GCRα reduction; hence the GCRα/GCRβ expression ratio rises. It seems that in cases of asthma and SRA, the observed discrepancies in GCRα and GCRβ expressions occurred due to pathogenesis and/or other interfering factors. Although there were some differences in sampling and methodology, our finding partly documents the study of Goleva *et al.*, (2012) who tried to introduce a marker for GC resistance in asthma. Our results were comparable with their findings that in PBMLs of SRA cases, the GCRβ expression increased while the GCRα/GCRβ expression ratio decreased (6). 

Using different methods, several studies on different airway diseases have showed controversies in GCRs expressions (33). An *in-vitro* study showed that corticosteroids down regulate GCRα expression in respiratory cell lines dose-dependently (15). So, our finding of lower expression of GCRα in PBMLs of the SRA cases was probably due to prescription of systemic GCs; although it does not explain low expression of GCRα in asthma cases who had not received systemic GCs. Regarding these findings and in contrast to the proposed role of GCRα in GC resistance (34), it seems that decrease in GCRα expression occurs in every asthma cases (both mild and severe), and its higher reduction in SRA cases may be due to systemic GC treatment. Thus, decline in GCRα expression is not the main cause of GC resistance; as it has been seen in non-severe forms as well. 

**Table 1:** Designed primers for amplification of GCRα, GCRβ and GAPDH

		**Primer sequence**	**Primer Length (BP)**	**Product length (BP)**
GCRα	F	5’-CTTACTGCTTCTCTCTTCAGTTCC-3’	24	193
	R	5’-GAGATTTTCAACCACTTCATGC-3’	22	
GCRβ	F	5’-CTTACTGCTTCTCTCTTCAGTTCC-3’	24	198
	R	5’-GGTTTTAACCACATAACATTTTCA-3’	24	
GAPDH	F	5’-CCATGAGAAGTATGACAAC-3’	19	115
	R	5’-GAGTCCTTCCACGATACC-3’	18	

**Table 2:** Demographic data of participants

	**Severe asthma**	**Asthma**	**Healthy**	**Total**	**Total ** ***P*** **-value**
**Gender**					
-Male	8	9	27	44	
-Female	5	5	3	13	
-Total	13	14	30	57	0.52
**Age (years)**					
-Mean ± SE	50.23 ± 3.80	54.71 ± 4.31	39.17 ± 1.54	45.51 ± 1.80	<0.001
-95% CI	41.95-58.51	45.39-64.4	36.01-42.33	41.89-49.13	
-Min.-Max.	21-76	24 -80	23-60	21-80	
**BMI (kg/M** ^2^ **)**					
-Mean ± SE	26.39 ± 1.25	26.41 ± 0.81	26.49 ± 0.70	26.45 ± 0.50	0.99
-95% CI	23.65-29.14	24.65-28.16	25.04-27.93	25.44-27.45	
-Min.-Max.	19.53-35.49	20.05-30.12	18.65-34.09	18.65-35.49	

**Table 3:** Data of GCRα and GCRβ gene expressions

	**GCRα (ΔCt)**					**GCRβ (ΔCt)**						
	**Mean ± SE**	**95% CI**	**Min.-Max.**		***P*** **-value**	**Mean ± SE**		**95% CI**		**Min.-Max.**		***P*** **-value**
**Gender**												
-Male	2.46 ± 0.16	2.12-2.79	0.63-5.01	0.77		11.72 ± 0.34	11.02-12.42		5.69-15.78		0.85	
-Female	2.57 ± 0.48	1.50-3.64	-0.32-5.83			11.58 ± 0.70	10.05-13.12		5.78-14.73			
**Groups**												
-Severe asthma	2.75 ± 0.42	1.82-3.68	-.032-4.71	0.65		11.55 ± 0.58	10.27-12.82		8.37-15.37		0.77	
-Asthma	2.47 ± 0.44	1.50-3.44	-0.12-5.83			12.09 ± 0.61	10.75-13.43		8.38-15.73			
-Healthy	2.37 ± 0.14	2.07-2.67	0.65-3.56			11.57 ± 0.45	10.63-12.51		5.69-15.78			
Total	2.48 ± 0.16	2.16-2.81	-0.32-5.83			11.69 ± 0.31	11.07-12.31		5.69-15.78			

**Table 4:** Correlation of GCRα and GCRβ gene expressions

		**Age** **r (** ***P*** **-value)**	**BMI** **r (** ***P*** **-value)**	**GCRα ΔCts** **r (** ***P*** **-value)**
Severe Asthma	Age	-		
	BMI	-0.125 (0.685)	-	
	GCRα ΔCts	-0.709^*^ (0.007)	-0.160 (0.602)	-
	GCRβ ΔCts	-0.081 (0.793)	0.072 (0.816)	0.533 (0.61)
Asthma	Age	-		
	BMI	0.083 (0.778)	-	
	GCRα ΔCts	-0.509 (0.063)	-0.246 (0.397)	-
	GCRβ ΔCts	-0.404 (0.171)	-0.232 (0.446)	0.786^*^ (0.001)
Healthy	Age	-		
	BMI	0.116 (0.542)	-	
	GCRα ΔCts	0.139 (0.464)	-0.043 (0.821)	-
	GCRβ ΔCts	0.257 (0.178)	-0.089 (0.646)	0.490^*^ (0.007)

* Correlation is significant at the 0.01 level (2-tailed).

On the other hand, Pujols *et al*. showed that in cell lines, GCs down regulate GCRβ in a short period of time (15) while we found the opposite in SRA; similar to what the others have shown (12, 30 and 31). Furthermore, there was down-regulation of GCRβ in asthmatics that were not on systemic GCs. Thus, it seems that in non-severe asthma, down-regulation of GCRα and GCRβ occurs, with a higher degree for GCRβ. But in SRA, systemic GC treatment decreases GCRα expression more, while expression of GCRβ increases; in contrast to the expected effect of GCs on the cell lines. These expression changes describe why the GCRα/GCRβ expression ratio increased in non-severe asthma and decreased in SRA; the finding which previously mentioned as a critical criterion for definition of GC sensitivity and resistance (12). Also, GC prescription cannot merely explain the mechanism(s) of GC resistance, unless other possible mechanisms (*e.g.* transcriptional, post-transcriptional and post-translational) are involved. It worth to be noted that we studied clinical expression where many factors can interfere. For example, the stability of GCR mRNA may be influenced by estrogens and iron. Also, some cytokines (*i.e*. *IL-2* and *IL-4*) can decrease GCRα expression without affecting GCRβ (15, 18).

The increase of GCRβ expression in SRA has been reported in some studies and many investigators believe that due to the negative effect of this isoform on GCRα activity, it may be a notable cause in SRA pathophysiology. This hypothesis has been proposed in other GC resistance conditions like leukemia, ulcerative colitis, and nasal polyps, too. (12). However, some authors doubt it and mention GCRβ as a very low-expressed intranuclear peptide (compared to GCRα); hence, it has less effect on GCRα activity (12, 35 and 36). Furthermore, as far as our knowledge extends, no evidence has been published so far to show either GCRβ o`vercome GCRα or its upregulation suppresses the anti-inflammatory actions of GCRα in clinical samples of SRA (33).

Similar to the study of Jakiela *et al.*, the current study showed that GCRβ is expressed hundreds times less than GCRα in all disease/health conditions (36); so logically, the beta isotype either has no role on GC resistance or should compete or affect GCRα potently, in order to influence its activities. Furthermore, the effect of GCRβ may be non-competitive or unknown. For instance, there is an evidence that GCRβ can disrupt GCRα nuclear translocation and it may constitute the underlying mechanism (35). This finding has been confirmed in smooth muscles of airways in severe asthma cases, too (37). As the other mechanism, it is also proposed that GCRβ controls the expression of histone deacetylase 2 through inhibiting GC response elements in its promoter (38), while it is not confirmed by the others (39).

The lack of correlation between GCRα and GCRβ in SRA (Table 4) has been supported by some studies. For example, the microarray study of Kino *et al*. indicated that GCRβ can implement intrinsic gene-specific transcriptional activity, in a GCRα independent way. The ability of GCRβ to negatively or positively regulate a large number of genes may be a hypothesis for its role in GC resistance (40). Also, some other GCRβ-related metabolic pathways may be responsible for SRA. For example, Vazquez-Tello *et al*. showed that some asthma-related cytokines (like *IL-17* and *IL-23*) can significantly increase GCRβ expression (18).

Thus, all together, it is not wise to consider GCRs as the main causes of GC resistance and the other proposed genetic and acquired factors (41) should be considered simultaneously. However, up-regulation of GCRβ in SRA is an actual finding, which has no relation to GC treatment. It seems that in collaboration with other molecular factors and mechanisms, GCRβ is related to GC resistance; however, it is not exactly clear whether or not increased GCRβ is a causative factor in SRA pathogenesis. Nevertheless, its very low expression level means that competence with GCRα is less likely and the other mechanisms are involved. Furthermore, it should be emphasized that increased GCRβ expression is just one of the several proposed mechanisms for GC resistance (42).

The current cross-sectional study was performed on PBMLs, which represent systemic data; however, different cells of the immune and respiratory systems are involved in the pathogenesis of asthma. Thus, the simultaneous expression study of the candidate genes at the pulmonary and immune levels will provide more comprehensive and applicable data in this regard. But according to ethical issues, it was not possible for us to use invasive procedures (like surgery or bronchoscopy) to obtain pulmonary samples. Furthermore, the restricted inclusion criteria made our sample size limited and it may be partly the reason of non-significant results. Furthermore, asthma and its severe form are complex multifactorial disorders, which have no unique definition. Thus, various sampling criteria and bioenvironmental factors may yield in different results of various studies. It seems that well-defined longitudinal comprehensive studies of clinical, molecular, and bioenvironmental factors with reasonable sample size will provide more valid and reliable results.

It should be noted that this study just assessed the GCR activity at the transcriptional level of SRA cases. While some studies at the proteomic level have supported our findings (43, 44), many other factors at the post-transcriptional and protein levels may change the expression results. So, comprehensive proteomics studies will provide more valuable information in this respect; although, it was not possible for us to perform that, technically.

Finally, we would like to re-emphasize that there are other isoforms of GCRs, which may play roles, especially when GCRα and GCR-γ expressions are inevitably evaluated 

altogether.

## Conclusion

The present *in-vivo* quantitative study indicates that GCRs are related to, but not directly responsible for SRA pathogenesis and their expression evaluation is not valuable for diagnosis of GC resistance. The lack of correlation and wide gap between GCRs’ expression in SRA show that GCRβ does not inhibit GCRα in a competitive manner. On the other hand, it seems that the reduction in GCRα/GCRβ expression ratio is a major notable finding, but not a causative factor, in GC resistance and further studies on its role in diagnosis or treatment of GC resistance cases may be useful. However, other causative and/or confounding factors and mechanisms (like medications, metabolic pathways, cytokines, hormones, minerals, *etc.*) most probably interfere with the complex pathogenesis of GC resistance in severe asthmatics.

## Supplementary Materials

Calculation of GCRα and GCRβ Gene Expressions BY 2-ΔΔCT Analysis Method Figures S1 to S3
