# Supplementary material for: Different Gene Expressions of Alpha and Beta Glucocorticoid Receptors in Asthmatics
Source: Iran J Pharm Res. 2018 Spring;17(2):790–800. (PMC5985195)
Supplement: Supplementary file 1 — Calculation of GCRα and GCRβ Gene Expressions BY 2-ΔΔCT Analysis Method Figures S1 to S3 [file ijpr-17-790-s001.pdf]

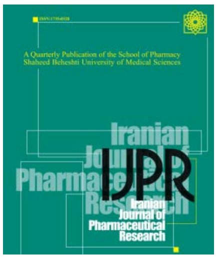

Supplementary Materials for

Different Gene Expressions of Alpha and Beta Glucocorticoid Receptors in Asthmatics

Kayvan Saeedfar, Mehrdad Behmanesh*, Esmaeil Mortaz and Mohammad Reza Masjedi

*To whom correspondence should be addressed. E-mail: behmanesh@modares.ac.ir

Volume 17, Issue 2 (Spring 2018)

This PDF file includes:

Calculation of GCRα and GCRβ Gene Expressions BY 2-ΔΔCT Analysis Method

Figures S1 to S3

Calculation of GCRα and GCRβ Gene Expressions
BY $2^{-\Delta\Delta CT}$ Analysis Method

Part 1

Relations of the GCRα expression among the groups:

Mean ΔCts of Asthma group: 2.47
Mean ΔCts of SRA group: 2.75
Mean ΔCts of Control group: 2.37
So,

1) Calculation of $2^{-\Delta\Delta Ct}$ for Asthma *vs.* Control: $2^{-(2.47-2.37)} = 2^{-0.1} = 0.93303$
2) Calculation of $2^{-\Delta\Delta Ct}$ for SRA *vs.* Control: $2^{-(2.75-2.37)} = 2^{-0.38} = 0.76843$
3) Calculation of $2^{-\Delta\Delta Ct}$ for SRA *vs.* asthma: $2^{-(2.75-2.47)} = 2^{-0.28} = 0.82359$

Relations of the GCRβ expression among the groups:

Mean ΔCts of Asthma group: 12.09
Mean ΔCts of SRA group: 11.55
Mean ΔCts of Control group: 11.57
So,

1) Calculation of $2^{-\Delta\Delta Ct}$ for Asthma *vs.* Control: $2^{-(12.09-11.57)} = 2^{-0.52} = 0.69737$
2) Calculation of $2^{-\Delta\Delta Ct}$ for SRA *vs.* Control: $2^{-(11.55-11.57)} = 2^{0.02} = 1.01395$
3) Calculation of $2^{-\Delta\Delta Ct}$ for SRA *vs.* asthma: $2^{-(11.55-12.09)} = 2^{0.54} = 1.45397$

Part 2

Relations of the GCRα and GCRβ expressions in each group:

In Asthma group;
Mean ΔCts of GCRα: 2.47
Mean ΔCts of GCRβ: 12.09
So,

1) Calculation of $2^{-\Delta\Delta Ct}$ for GCRα *vs.* GCRβ: $2^{-(2.47-12.09)} = 2^{9.62} = 768.8800$
2) Calculation of $2^{-\Delta\Delta Ct}$ for GCRβ *vs.* GCRα : $2^{-(12.09-2.47)} = 2^{-9.62} = 0.0012$

In SRA group;
Mean ΔCts of GCRα: 2.75
Mean ΔCts of GCRβ: 11.55
So,

1) Calculation of $2^{-\Delta\Delta Ct}$ for GCRα *vs.* GCRβ: $2^{-(2.75-11.55)} = 2^{8.8} = 445.7218$
2) Calculation of $2^{-\Delta\Delta Ct}$ for GCRβ *vs.* GCRα : $2^{-(11.55-2.75)} = 2^{-8.8} = 0.0022$

In control group;
Mean ΔCts of GCRα: 2.37
Mean ΔCts of GCRβ: 11.57
So,

1) Calculation of $2^{-\Delta\Delta Ct}$ for GCRα *vs.* GCRβ: $2^{-(2.37-11.57)} = 2^{9.2} = 588.1335$
2) Calculation of $2^{-\Delta\Delta Ct}$ for GCRβ *vs.* GCRα : $2^{-(11.57-2.37)} = 2^{-9.2} = 0.0017$

The Dot Plot Diagram of Correlations of the GCRα and GCRβ Gene Expression

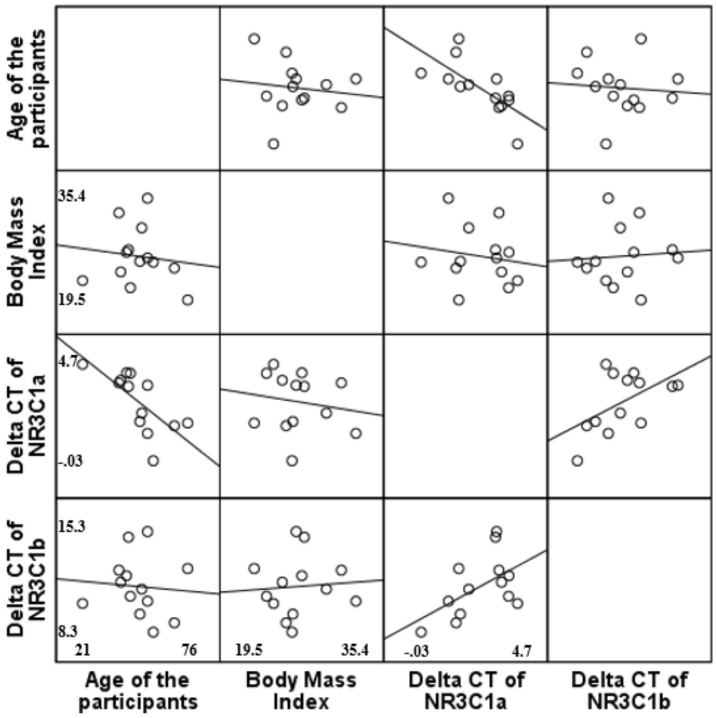

Figure S1. Dot plot diagram of correlation in severe asthma group (Age and BMI are presented in year and kg/M^2 respectively. Delta CTs are ratio, have no unit).

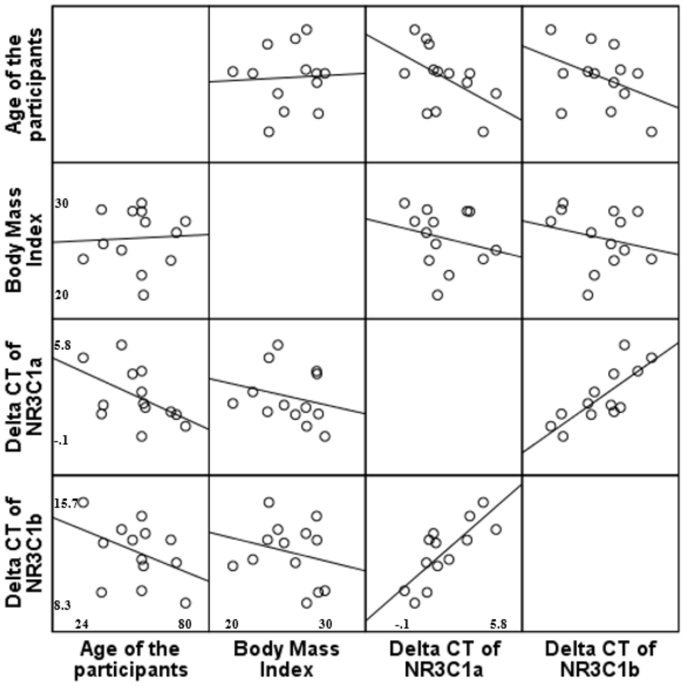

Figure S2. Dot plot diagram of correlation in asthma group (Age and BMI are presented in year and kg/M^2 respectively. Delta CTs are ratio, have no unit).

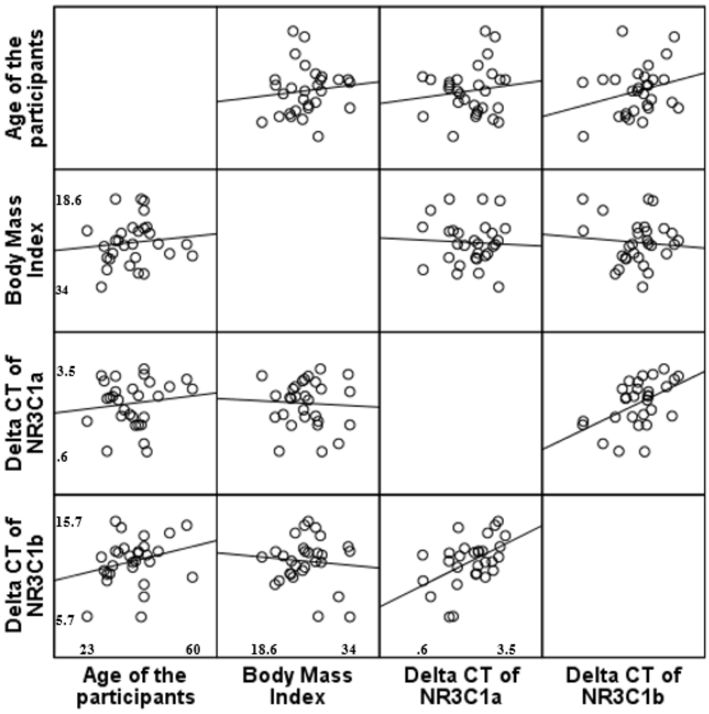

Figure S3. Dot plot diagram of correlation in control group (Age and BMI are presented in year and kg/M^2 respectively. Delta CTs are ratio, have no unit).
